# Supplementary material for: Collaborative intelligence in AI: Evaluating the performance of a council of AIs on the USMLE
Source: PLOS Digit Health. 2025 Oct 9;4(10):e0000787. doi: 10.1371/journal.pdig.0000787 (PMC12510544; doi:10.1371/journal.pdig.0000787)
Supplement: S2 File — (ZIP) [file pdig.0000787.s002.zip › S2 Data Raw data files containing unprocessed question inputs and LLM outputs including transcripts of deliberations/STEP3_Question-53-1_Type-MC-NJ.docx]

**Facilitator** **[Round 1]:** In determining the usefulness of a D-dimer assay in ruling out pulmonary embolism as a diagnosis in a 27-year-old woman with pleuritic chest pain and nonproductive cough, a recent study in the medical literature is reviewed. The study evaluates the use of D-dimer assay in 1500 patients (705 female and 795 male) ranging in age from 19 years to 57 years (mean age is 36 years), whose examining physicians had a low clinical suspicion that the patients had pulmonary embolism. All patients in the study underwent a D-dimer assay, followed by CT angiography of the chest to confirm or exclude the diagnosis of pulmonary embolism. Study results are shown: D-Dimer Positive and CT Angiography Positive: 88 D-Dimer Negative and CT Angiography Positive: 0 D-Dimer Positive and CT Angiography Negative: 312 D-Dimer Negative and CT Angiography Negative: 1100 Total CT Angiography Positive: 88 Total CT Angiography Negative: 1412 Total D-Dimer Positive: 400 Total D-Dimer Negative: 1100 Which of the following is the most appropriate conclusion about this data in regard to patients with low clinical suspicion for pulmonary embolism? (A) D-Dimer assay results should be used to determine the need for ordering CT angiography for patients suspected of having a pulmonary embolism (B) False-negative D-dimer assay results are common in patients with confirmed pulmonary embolism (C) False-positive D-dimer assay results are rarely encountered (D) A negative D-dimer assay has a high negative predictive value for excluding the diagnosis of pulmonary embolism (E) A positive D-Dimer assay has a high positive predictive value for diagnosing pulmonary embolism

**AI_A:** RESTATING THE QUESTION: The question seeks the best conclusion regarding the use of a D-dimer assay in diagnosing pulmonary embolism, particularly in patients with a low clinical suspicion of having this condition, based on the presented study data. A D-dimer assay is a test that measures the presence of D-dimer, a small protein fragment present in the blood after a blood clot is degraded by fibrinolysis. Pulmonary embolism is a condition characterized by a sudden blockage in the lung artery, usually due to a blood clot that has traveled from elsewhere in the body, frequently the leg. PLANNING: Some assumptions in this case include that Positive D-dimer assay results indicate the presence of a clot (and hence the possibility of pulmonary embolism), and Negative D-dimer assay results suggest the absence of a clot (thus lowering the probability of pulmonary embolism). The assumption to question further due to high clinical significance would be the accuracy and efficiency of the D-dimer assay, considering its false-positive and false-negative rates. CLINICAL REASONING: The D-dimer assay had falsely identified the presence of clots in 312 cases (D-Dimer Positive and CT Angiography Negative), which means that it has a substantial rate of false-positive results. However, it failed to miss any actual cases of pulmonary embolism (D-Dimer Negative and CT Angiography Positive cases are 0), suggesting that the D-dimer assay has a small or non-existent false-negative rate. From this data, it's apparent that a Negative D-dimer result is considerably reliable in ruling out a pulmonary embolism. CONCLUDING: A conclusive interpretation of this data shows that the D-dimer assay's Negative Predictive Value (the probability that subjects with a negative screening test truly don't have the disease) in this population seems high. The assay rarely gives false-negative results, while false-positives are more common. Therefore, a negative D-dimer assay is reasonably dependable in ruling out a diagnosis of pulmonary embolism, particularly in patients with a low clinical suspicion for the condition. RESPONSE: (D) A negative D-dimer assay has a high negative predictive value for excluding the diagnosis of pulmonary embolism.

**AI_C:** RESTATING THE QUESTION: The question queries on how to best apply the data provided from a recent medical study on the use of D-dimer assays for diagnosing pulmonary embolism (PE), particularly in patients with low clinical suspicion for PE. A D-dimer assay, an addition to standard laboratory testing, measures the presence of D-dimer in your body's blood, a substance that's often present in higher amounts when there are blood clots, like a PE. An assumption to question is whether a positive D-dimer test indicates a high probability of having a PE. PLANNING: Alternate assumptions to generate include whether a negative D-dimer assay has a high value for excluding PE or if D-dimer assays should be used to determine the need for more specific testing like a CT angiography. Evaluating these assumptions, it would seem plausible to focus on the predictive values of the D-dimer test. CLINICAL REASONING: A high negative predictive value (NPV) would mean that a negative result on a D-dimer test indicates a very low probability of having a PE. This would be useful to confidently exclude the diagnosis in low suspicion patients, reducing the need for further invasive and expensive testing. Looking at the data, there were no patients with a negative D-dimer and a positive CT angiography. This appears to be a great support for this hypothesis, as there were no false negatives. CONCLUDING: Using the clinical thought that a high NPV is extremely useful in a rule-out test, the data shows that the D-dimer assay has a high NPV, thus it aligns with the goal of using this test primarily as a rule-out method for PE in patients with a low clinical suspicion for this condition. RESPONSE: Based on the evidence provided in the study's results and utilising principles of diagnostic decision-making, the conclusion is that a negative D-dimer assay has a high negative predictive value for ruling out the diagnosis of pulmonary embolism (Choice D). Therefore, this becomes the most appropriate conclusion about the data in regard to patients with low clinical suspicion for pulmonary embolism.

**AI_D:** RESTATING THE QUESTION: We need to extract meaning from the provided data, which involves the results of D-dimer tests and CT angiography of the chest in patients with low clinical suspicion for a pulmonary embolism (PE). The D-dimer test is a blood test that can be used to help check for or monitor blood clotting problems. A positive D-dimer result may suggest that there has been significant blood clot (thrombus) formation and breakdown in the body, but it does not tell the location or cause. Conversely, a CT angiography positive indicates the presence of a PE. The question is centered around the appropriateness of the conclusion drawn about the data, specifically in relation to the diagnostic value of the D-dimer assay. PLANNING: Upon examining the data, we can see that no patients had a positive CT angiography but a negative D-dimer (i.e., no false negatives) while there were several false positives (D-dimer positive, CT angiography negative). To reach a cogent conclusion regarding the D-dimer test, we need to question assumptions about the diagnostic value of the test and particularly the prevalence of negative and positive predictive values. CLINICAL REASONING: A high negative predictive value (NPV) means that when the test result is negative, it is highly likely that the patient truly does not have the disease. In this study, all patients who tested negative for D-dimer also did not show signs of PE upon CT angiography. Hence, the NPV of D-dimer assay seems to be high. The positive predictive value (PPV), on the other hand, denotes the probability that subjects with a positive test truly have the disease. As 312 out of 400 patients with positive D-dimer results were CT angiography-negative, this test does not seem to have a high PPV. CONCLUDING: In light of the observation, the conclusion about the D-dimer assay that can be drawn in patients with low clinical suspicion for PE is that a negative D-dimer assay has a high negative predictive value for PE, but a positive result does not accurately predict the presence of the disease due to a significant number of false-positive results. RESPONSE: Hence the answer is (D) A negative D-dimer assay has a high negative predictive value for excluding the diagnosis of pulmonary embolism. This means that a negative D-dimer test result is a good indication that the person does not have an acute condition causing the formation of blood clots. However, positive D-dimer test results cannot conclusively indicate the presence of a PE due to the relatively high incidence of false positives.

**AI_E:** (1) RESTATING THE QUESTION: We're discussing the role of the D-dimer assay in diagnosing pulmonary embolism, particularly in a clinical scenario where the suspicion of this condition is low. The D-dimer assay measures the amount of D-dimer in the blood, which increases in the presence of a clot. Thus, it can be used to rule out conditions like a deep vein thrombosis or pulmonary embolism. The study has analyzed patients with low clinical suspicion for pulmonary embolism who underwent both D-dimer assay and CT angiography - a definitive diagnostic tool. From the given data, we need to draw conclusions about the effectiveness and reliability of the D-dimer assay. (2) PLANNING: We need to calculate the negative predictive value (the probability that a negative test accurately indicates absence of a condition) and the false-positive rate (the probability that a positive test inaccurately indicates presence of a condition) for the D-dimer assay. (3) CLINICAL REASONING: The study data shows us that: - Number of D-dimer negatives that are actually free of pulmonary embolism (true negatives) = 1100. - Number of D-dimer positives that actually have pulmonary embolism (true positives) = 88. - Number of D-dimer positives that do not actually have pulmonary embolism (false positives) = 312. The fact that there were zero D-dimer negatives who had pulmonary embolism (false negatives) implies that the test indeed may have a high negative predictive value. (4) CONCLUDING: Negative Predictive Value (NPV) can be determined by the formula: NPV = True Negatives / (True Negatives + False Negatives). Given that there were no false negatives, the NPV comes out to be 1 or 100%, representing a high negative predictive value. This indicates that a negative D-dimer result successfully excludes a diagnosis of pulmonary embolism. However, the significant number of false-positive D-dimer results suggests that a positive D-dimer assay does not strongly predict the presence of pulmonary embolism. (5) RESPONSE: The most appropriate conclusion based on the given data would be that a negative D-dimer assay has a high negative predictive value for excluding the diagnosis of pulmonary embolism (Option D). It is effective in ruling out pulmonary embolism when suspicion is low, but a positive result should not be relied upon without further confirmation due to its high false-positive rate.

**AI_B:** RESTATING THE QUESTION: The question asks for the most appropriate conclusion about the presented data, pertaining to patients with a low clinical suspicion for pulmonary embolism. The data come from a study that used both a D-Dimer assay and CT angiography to diagnose or rule out pulmonary embolism in 1500 patients. The D-Dimer assay is a blood test that measures the presence of D-Dimer, a protein fragment produced when a blood clot dissolves. CT angiography is a radiological test that uses contrast dye and X-rays to visualize blood flow in pulmonary arteries. PLANNING: The assumptions made by each answer choice are: (A) D-Dimer assay results should decide whether to perform CT angiography. (B) There are many false-negative D-dimer assay results among patients with confirmed pulmonary embolism. (C) False-positive D-dimer assay results are seldom seen. (D) A negative D-dimer assay is highly likely to accurately rule out a diagnosis of pulmonary embolism (high Negative Predictive Value). (E) A positive D-Dimer assay is highly likely to accurately confirm a diagnosis of pulmonary embolism (high Positive Predictive Value). Assumptions B and E can be rejected because, according to the results, there were no false negatives (B is false), and there were far more false positives (312) than true positives (88), meaning E is also false. Options A and C require a judgment that can be clinically scrutinized using the data. However, option D seems the most promising assumption given the zero number of false negatives and a large number of true negatives (1100). CLINICAL REASONING: The data can be analyzed using concepts of medical statistics primarily sensitivity, specificity, and predictive values. Sensitivity refers to how good the test is at identifying a disease when it is present, and specificity refers to how good it is avoiding false positives. Predictive values tell you what the probability is that the disease is present (positive predictive value, or PPV) or absent (negative predictive value, NPV) given the test result. The lack of false negatives (Patients who tested negative, but actually had the disease), in this case, means that D-dimer assay showed perfect sensitivity. However, specificity was not excellent given the numerous false positives (Positive test but no disease), calling into question assertion A about using the D-dimer assay to decide on further diagnostic steps. Therefore, the high number of true negatives (negative test and no disease) and nil false negatives enhance its Negative Predictive Value (NPV, calculated as true negatives/true negatives + false negatives). This rendering assertion D as the only viable option. CONCLUDING: Given the evidence, a D-Dimer assay has a high exclusionary value for pulmonary embolism among patients who are clinically regarded as having a low suspicion for the condition. The large number of true negatives and the absence of false negatives make it reliable in ruling out the disease but less so in definitely confirming it due to lower specificity marked by a higher rate of false positives. RESPONSE: Therefore, the most appropriate conclusion about this data in regard to patients with low clinical suspicion for pulmonary embolism is choice D, which states that a negative D-dimer assay has a high negative predictive value for excluding the diagnosis of pulmonary embolism.

**Facilitator** The question seeks to understand the diagnostic value of a D-dimer assay in ruling out pulmonary embolism, particularly in patients with low clinical suspicion for the condition. The D-dimer assay is a blood test that measures the presence of D-dimer, a protein fragment produced when a blood clot dissolves. The data from the study indicates that there were no false negatives (patients who tested negative for D-dimer but had pulmonary embolism confirmed by CT angiography), suggesting that the D-dimer assay has a high negative predictive value. This means that a negative D-dimer result is highly likely to accurately indicate the absence of pulmonary embolism. However, the study also showed a significant number of false positives (patients who tested positive for D-dimer but did not have pulmonary embolism confirmed by CT angiography), indicating that a positive D-dimer result does not strongly predict the presence of pulmonary embolism. Therefore, while a negative D-dimer assay is reliable in ruling out a diagnosis of pulmonary embolism, particularly in patients with a low clinical suspicion for the condition, a positive result should not be relied upon without further confirmation due to its high false-positive rate. In conclusion, the most appropriate interpretation of the data is that a negative D-dimer assay has a high negative predictive value for excluding the diagnosis of pulmonary embolism in patients with low clinical suspicion for the condition.
